# Breast cancer: The level of stress correlated with the type of surgery and the instructive level of patients

**DOI:** 10.1192/j.eurpsy.2021.1160

**Published:** 2021-08-13

**Authors:** T. Catalina Dana, C. Giurgi-Oncu, G. Daciana, O. Cristina, S. Ica, I. Rivis, N. Adriana, D. Bianca, A. Bredicean

**Affiliations:** 1 Psychiatry I, “PIUS BRINZEU” COUNTY EMERGENCY HOSPITAL, PSYCHIATRIC CLINIC “EDUARD PAMFIL”, TIMISOARA, Romania; 2 Psychiatry, “Victor Babes” University of Medicine and Pharmacy, Timisoara, Romania; 3 Plastic And Reconstructive Surgery, “Victor Babes” University of Medicine and Pharmacy, Timisoara, Romania; 4 Medical Oncology, ONCOHELP MEDICAL CENTER, TIMISOARA, Romania; 5 Plastic And Reconstructive Surgery, “PIUS BRINZEU” COUNTY EMERGENCY HOSPITAL, TIMISOARA, Romania; 6 Neurosciences, “Carol Davila” University of Medicine and Pharmacy, Bucharest, Romania; 7 Psychiatry, “DR.VICTOR POPESCU” EMERGENCY MIITARY HOSPITAL, TIMISOARA, Romania; 8 Neurosciences, “Victor Babes” University of Medicine and Pharmacy, Timisoara, Romania

**Keywords:** BREAST-CANCER, surgery, stress, Education

## Abstract

**Introduction:**

Breast cancer is a severe pathology that once detected completely changes the patient’s perception of life.

**Objectives:**

Evaluating the relationship that is established between the level of stress, the type of surgery applied and, the instructive level of women.

**Methods:**

We selected 67 patients which were divided into 2 groups: group I(31) women who benefited from immediate reconstruction and group II(36) subjects who benefited from a late reconstruction. We split each group into two subgroups: women with secondary education and women with higher education. A socio-demographic questionnaire and the DASS-21 scale were applied.

**Results:**

Comparing the two groups we noticed that stress level was more present in group I(38,7%) than in group II(25%). The differences were not statistically significant(p>0,05). In the subgroup of women with higher education in group I, high levels of stress were observed at 23,08% and, in the subgroup of patients with secondary education, 50% had high levels of stress. The differences were not statistically significant (p>0,05). We also analyzed the two subgroups of group II and we identified increased levels of stress in 20% of patients with higher education compared to those with secondary education where 26,93% had high levels of stress. Also, the differences were not statistically significant(p>0,05). A statistically significant difference(p<0,05) was found when we compared the level of stress between women with secondary education of group I and those of group II.

**Conclusions:**

The study revealed that stress levels tend to be higher in women with immediate breast reconstruction and secondary education.

